# One-Year and Five-Year Outcomes in Medication Overuse Headache: A Real-World Study

**DOI:** 10.7759/cureus.72347

**Published:** 2024-10-25

**Authors:** Abouch Krymchantowski, Carla Jevoux, Ana Gabriela Krymchantowski, Rogelio Dominguez-Moreno, Raimundo Pereira Silva-Néto

**Affiliations:** 1 Department of Neurology, Headache Center of Rio, Rio de Janeiro, BRA; 2 Department of Neurology and Psychiatry, Instituto Nacional De Ciencias Médicas Y Nutrición "Salvador Zubirán", Mexico City, MEX; 3 Department of Neurology, Universidade Federal do Delta do Parnaíba, Parnaíba, BRA

**Keywords:** chronic migraine, headache days per month, medication overuse headache, monoclonal antibody, preventive treatment

## Abstract

Background: Medication overuse headache (MOH) is one of the global health-related problems that imposes significant morbidity. Effective management requires the abrupt cessation of the overused medications, transition therapy in the initial days, and initiation of preventive treatment. The objective of this study is to provide one-year and five-year follow-ups of study participants diagnosed with chronic migraine and MOH. The study will examine the efficacy of withdrawal therapy, the use of conventional preventive medication, and the use of anti-calcitonin gene-related peptide (anti-CGRP) monoclonal antibodies.

Methodology: We conducted a single-center, prospective, and descriptive study at a tertiary center in Brazil. The population was included by convenience sampling of consecutive subjects diagnosed with chronic migraine and MOH. Demographics and clinical data at baseline and one-year and five-year follow-ups were collected in the clinical records.

Results: Among 142 subjects, 116 were females and 26 were males, with a mean age of 42.1±14.3. They were followed for five years. The diagnosis was performed at the mean age of 24.9±14.7 years after the headache onset, and the time with headache ≥15 days per month was 6.3±7.6 years. On baseline, the average number of headache days per month (HDM) was 25.2±5.9. There was a reduction in HDM. At one-year and five-year follow-ups, a ≥75% reduction in HDM was observed, respectively, in 51.4% and 70.4% of the sample.

Conclusions: The five-year follow-up of chronic migraine and MOH treated with the discontinuation of excessive medication, the use of preventative pharmacological agents, and the optional inclusion of anti-CGRP pathway monoclonal antibody led to a significant decrease in the initial occurrence of HDM.

## Introduction

Medication overuse headache (MOH) is a prevalent debilitating condition frequently encountered in neurological practice, affecting 1-7% of the global population and impacting over 60 million individuals [1-4]. The burden of MOH is significant, impairing work productivity and quality of life, with comorbid sleep and psychiatric disturbances further exacerbating the suffering and healthcare costs [1,2,5].

MOH arises from excessive drugs for acute treatment, often seen in patients with preexisting migraine or tension-type headaches [4,6]. The variability in overuse patterns and the pathophysiological mechanisms underlying MOH progression remain areas of active research and debate [7-11]. Despite these uncertainties, it is widely recognized that MOH primarily affects individuals with primary headache disorders [12-17].

Treating MOH is challenging, with no consensus on the best approach, particularly across different geographic realities [18-26]. Effective treatment strategies are limited by patients' adherence and varying responses, underscoring the need for comprehensive and individualized approaches [4,5,19]. The sudden and complete withdrawal of overused medications is considered a cornerstone of MOH management, though this approach has faced recent challenges [23,24]. Close follow-up with multidisciplinary support can reduce headache frequency reduction without necessarily initiating preventive medications, although bridge or transition therapies may be needed in cases involving certain drug overuse [4,9,22,25,27-29].

The initiation of preventive pharmacological agents following withdrawal remains controversial, with some evidence supporting their use to improve outcomes, including anti-calcitonin gene-related peptide (anti-CGRP) monoclonal antibodies (mAbs) [22,28-30]. However, long-term headache frequency reduction and the risk of relapse into medication overuse are not well understood. Combining medication withdrawal with preventive treatment appears to be the most effective strategy [4,9,25,26,31,32].

This study aimed to address these knowledge gaps by following consecutive patients with chronic migraine and MOH over five years. We observed the effectiveness of a comprehensive treatment approach involving medication withdrawal, traditional preventive agents, and the addition of anti-CGRP mAbs. Conducted in a Brazilian tertiary headache center, this long-term study provides valuable real-world data and insights into effective MOH management strategies.

## Materials and methods

Study design

This was an observational, prospective, uncontrolled, and descriptive study. The study population comprised a non-random sampling, consisting of consecutive patients diagnosed with chronic migraine and MOH treated at one tertiary clinic for five years. This study was performed after receiving approval from the Ethics in Research Involving Human Subjects Committee at the Federal University of Piauí with protocol number 3,305,167 and the National Ethics in Research System with registry number 08850918.0.0000.5214. This study recruited patients from the Headache Center of Rio, a tertiary center in Brazil, from August 2018 to July 2019.

Inclusion criteria

Patients aged 18 and above diagnosed with chronic migraine (defined as having 15 headache days per month (HDM) with at least eight days showing typical migraine symptoms) and MOH (defined as 15 HDM along with 10 or 15 days of symptomatic medication use for at least three months) were observed consecutively from July 2018 to July 2019 according to the International Classification of Headache Disorders (ICHD)-3 criteria [6].

Exclusion criteria

This study excluded individuals who had received botulinum toxin treatment in the past six months, those who had utilized traditional pharmacological agents for migraine prevention within the last three months, or patients with detectable psychiatric comorbidities aside from anxiety or unmedicated depression during the initial consultation. Furthermore, individuals who are pregnant or not utilizing effective contraceptive methods, as well as those intending to conceive within the next 12 months, were also excluded from the study.

Data collection

The informed consent was obtained from the participants who fulfilled the criteria for inclusion and exclusion. The diagnosis of chronic migraine and MOH was established, taking into account the frequency of headache episodes reported over the preceding three months.

Patients were clearly and emphatically oriented, with verbal and written instructions, to withdraw the excessive use of medications for headache attacks. They received the prescription, in up to two days a week, of either rizatriptan or zolmitriptan, associated with a nonsteroidal anti-inflammatory drug (NSAID). They were also prescribed migraine prophylactic drugs, either in monotherapy or in combination. The entire patient population received an objective headache diary to be filled out and presented during the follow-up visits, with intervals programmed and explained. The choice of preventive medication was based on the clinical experience of the attending physician, the history of previous use and/or failure of various pharmacological agents, as well as the possible presence of comorbidities.

Those patients who overused analgesics combined with caffeine, ergots, or both had to withdraw, received the prescription of indomethacin for the initial five days (50 mg twice a day), and, from the sixth day onwards, were started on preventive treatment. They were allowed to take rizatriptan or zolmitriptan plus an NSAID on a maximum frequency of two days per week. Those patients overusing triptans also had to withdraw, received the prescription of prednisone during the initial six days, and, from the seventh day onwards, started preventive treatment. These patients were also prescribed a triptan (rizatriptan or zolmitriptan) plus NSAID on a maximum frequency of twice a week, regardless of the previous overuse of a triptan. Collectively, studied patients withdrew, received bridge therapy of either indomethacin for five days or prednisone for six days depending on the previously used symptomatic medications, and started preventive treatments from the sixth or the seventh day onwards. During follow-up, from the 12th to the 24th months, those patients who did not experience a ≥75% reduction in headache frequency had the additional prescription of a mAb to the oral treatment.

Follow-up visits were conducted by the same attending physician bi-monthly for the initial four months, subsequently every four months for the next 12 months, and then every six months for the subsequent 44 months, totaling five years. The results were evaluated using headache diaries and patient information.

Statistical analysis

PASW Statistics for Windows, Version 18.0 (Released 2009; SPSS Inc., Chicago, Illinois, United States) was utilized for the purpose of statistical analysis. The quantitative variables were represented by their mean, standard deviation, and minimum and maximum values, whereas the qualitative variables were detailed through absolute and relative frequencies.

## Results

Two hundred and forty-three consecutive chronic migraine and MOH patients were evaluated during the studied period. Among them, 200 patients (161 females and 39 males) met the inclusion criteria and were included in the study. From the first follow-up visit, at two months onwards, 29% (58/200) of patients did not return for subsequent consultations and were lost to follow-up. They were excluded from the trial. The sample under investigation comprised 142 patients, with 81.7% (116 out of 142) being women and 18.3% (26 out of 142) men. The mean age was 42.1±14.3 years, with a range of ages from 18 to 84 years. The diagnosis was established 24.9±14.7 years after the headache was first experienced, ranging from two to 61 years. Time with headaches lasting more than 15 days per month was 6.3±7.6 years, ranging from one to 40 years (Table 1).

**Table 1:** Clinical and epidemiological characteristics of 142 patients with chronic migraine and medication overuse headache SD: standard deviation

Variables	Frequency
Sex	
Female (n; %)	116 (81.7%)
Male (n; %)	26 (18.3%)
Age at diagnosis (years)	
Average (mean±SD)	42.1±14.3
Variation	18-84
Time with headache (years)	
Average (mean±SD)	24.9±14.7
Variation	2-61
Time with headache >15 days/months (years)	
Average (mean±SD)	6.3±7.6
Variation	1-40

At the beginning of treatment, the average number of headache days was 25.2±5.9 per month, ranging from 16 to 30 days. The patients were followed for 60 months or five years. There was a reduction in the number of headache attacks per month, especially during the first year. At 12 months, the average headache frequency decreased to 7.6±6.1, representing a ≥75% reduction, observed in 51.4% (73/142) of the studied sample. At the end of follow-up, headache frequency was 5.7±4.1 days per month, a reduction of ≥75% in 70.4% of the patients (100/142). The incidence of headache days for the whole follow-up period is presented in Table 2 and Table 3, expressed as a percentage.

**Table 2:** Number of headache attacks per month during follow-up of 142 patients with chronic migraine and medication overuse headache SD: standard deviation

S. no.	Assessment periods (months)	Frequency (mean±SD)	Variation
1	Baseline	25.2±5.9	16-30
2	At 2	12.0±7.9	1-30
3	At 4	8.2±6.1	1-30
4	At 8	7.1±5.1	1-29
5	At 12	7.6±6.1	0-30
6	At 18	6.7±4.8	1-30
7	At 24	6.7±4.9	1-30
8	At 30	6.3±4.4	1-29
9	At 36	6.4±4.3	1-30
10	At 42	6.4±4.5	1-30
11	At 48	6.4±5.0	0-30
12	At 54	6.4±3.9	2-30
13	At 60	5.7±4.1	1-30

**Table 3:** Reduction in the average monthly headache days in the 12th and 60th months of treatment in 142 patients with chronic migraine and medication overuse headache

Percentage improvement	At 12 months		At 60 months	
	n	%	n	%
<50%	18	12.7	6	4.2
50-74%	51	35.9	36	25.3
≥75%	73	51.4	100	70.4

All patients were initially treated with oral medications, generally as a combination of pharmacological agents with one or two drugs. The drugs used, in order of frequency, were amitriptyline and atenolol (2.8%), divalproex sodium (3.5%), divalproex sodium and tizanidine (5.6%), topiramate and nortriptyline (14.8%), nortriptyline and flunarizine (26.8%), and nortriptyline, flunarizine, and tizanidine (46.5%). Of the 142 patients treated with oral medications, 57 (40.1%) added an anti-CGRP mAb, which started at some time during their treatment, no less than two years after the initial treatment phase, as shown in Table 4.

**Table 4:** Preventive treatment used in 142 patients with chronic migraine and medication overuse headache

Drugs	Frequency (n, %)
Oral medications (n=142)	
Amitriptyline and atenolol	4 (2.8%)
Divalproex sodium	5 (3.5%)
Divalproex sodium and tizanidine	8 (5.6%)
Topiramate and nortriptyline	21 (14.8%)
Nortriptyline and flunarizine	38 (26.8%)
Nortriptyline, flunarizine, and tizanidine	66 (46.5%)
Monoclonal antibodies (n=57/142; 40.1%)	
Erenumab (Pasurta®)	8 (14%)
Galcanezumab (Emgality®)	31 (54.4%)
Fremanezumab (Ajovy®)	18 (31.6%)

The specific combinations of nortriptyline, flunarizine, or nortriptyline, flunarizine, and tizanidine were compounded in a single capsule to be taken daily, during dinner. To prevent tolerability issues, the maximum daily dose of flunarizine was 2 mg, nortriptyline was 25 mg, and tizanidine was 5 mg. The decision was made based on the treating physician's extensive clinical experience gained from prescribing these medications over decades.

## Discussion

The treatment of MOH varies widely, influenced by different geographic realities [19,22,31]. This study followed chronic migraine and MOH patients over the long term at a specialized center in Brazil. Although the self-reported baseline headache frequency and symptomatic drug use may introduce bias, the findings offer important insights into the management of MOH in this setting.

The positive findings of this study, notably the significant reduction in headache frequency over five years, likely reflect a comprehensive treatment approach. This included long initial consultations, emphasis on stopping overused medications, and adherence to treatment strategies. A comparable study treated MOH sufferers with an outpatient detoxification program, achieving a two-thirds reduction in medication overuse within six months [26]. Another study revealed a 50% decrease in the frequency of headaches in almost half of the participants after a full year [27,32]. Our study observed a similar reduction at two months, but a greater decrease at 12 months and beyond, with a >75% reduction in nearly 52% of subjects at 12 months and even higher at 60 months.

While withdrawal of overused medication is the primary management for MOH, the best approaches also include patient education, psychological support, and starting preventive treatments for the underlying primary headaches. Initial bridge therapy may help reduce headache escalation and adherence issues [9,22,28,33]. Early and sudden interruption of symptomatic medications in outpatient settings, along with immediate preventive treatment, shows superiority [25,26,32,34,35]. However, inpatient strategies may be necessary for overuse involving opioids, barbiturates, or benzodiazepines [9].

Relapse is a common concern post-withdrawal, as chronic migraine reverts to an episodic form [8,18,22,27,31]. Few studies evaluate long-term outcomes and real-world relapse rates, highlighting the usefulness of this trial despite striking differences in healthcare settings [22,28,36,37]. For example, public health services in Brazil rarely have the infrastructure for long-term patient follow-up, while high-standard private centers offer updated management but may face cost limitations. This study's strength lies in its complete data over time, showing that withdrawal, support, and prevention significantly reduce headache frequency, especially in the first four months [22,38,39].

Limitations of the study

The study also acknowledged limitations, such as the potential bias in the studied population, which included highly motivated subjects treated at a specialized center. We did not assess particular characteristics such as severity scores, pain intensity, and the prevalence of cutaneous allodynia. Psychiatric comorbidities and substance use disorders were not scrutinized, which might affect treatment responses and adherence. Additionally, we did not compare outcomes between patients overusing different pharmacological classes, although benzodiazepine use was low, and none used opioids or barbiturates. Regarding anti-CGRP mAbs, 40% of the studied patients added mAbs to their regimen after a few years of stable treatment. This study did not evaluate the performance of mAbs compared to traditional agents, but initiating prevention with both traditional agents and adding a mAb led to higher headache frequency decreases regardless of the mAb used. Future studies should present data on preventives used across timeframes to better understand long-term adherence and outcomes.

## Conclusions

While evidence on the best MOH treatment is limited, starting prevention early alongside discontinuation of overused symptomatic treatments yields more favorable outcomes than withdrawal alone. Although combining medications with anti-CGRP mAbs may be superior to monotherapy, more randomized controlled trials are needed to evaluate the safety and efficacy of various preventive regimens. This prospective study of MOH patients at a Brazilian tertiary headache center enrolled 200 patients in a comprehensive treatment program. The program included clear orientation, sudden medication interruption, bridging medications for withdrawal, and preventive treatments, including anti-CGRP mAbs added after two years to improve adherence. The study demonstrated positive outcomes regarding patient adherence and headache reduction, underscoring the effectiveness of a structured and supportive treatment approach. However, the specific impact of adding mAbs remains inconclusive and warrants further investigation.
